# Is it the art or the artist?

**DOI:** 10.1016/j.xjon.2023.03.011

**Published:** 2023-04-05

**Authors:** Yung-Szu Wu, Ravi Ghatanatti, Joseph Zacharias

**Affiliations:** a Division of Cardiovascular Surgery, Cardiovascular Centre, Taichung Veterans General Hospital, Taichung City, Taiwan; b Cardiothoracic Surgery Department, Blackpool Victoria Hospital, Lancashire, United Kingdom

To the Editor:

**Figure:**
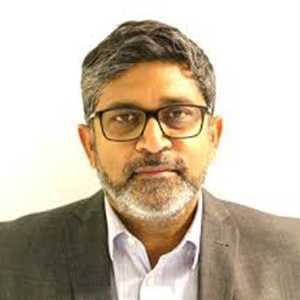
Joseph Zacharias, MD

J.Z. has recieved speaker and/or consulting fees from Edward lifesciences, Medtronic, Cryolife, Cambridge medical robotics, Terumo, and Ethicon in the past 36 months. All other authors reported no conflicts of interest.

The *Journal* policy requires editors and reviewers to disclose conflicts of interest and to decline handling or reviewing manuscripts for which they may have a conflict of interest. The editors and reviewers of this article have no conflicts of interest.

We read with interest the paper by Bonacchi and colleagues,^1^ who conducted a retrospective study to compare the outcomes of right anterior mini-thoracotomy and mini-sternotomy (MS) for minimally invasive aortic valve replacement. The study aimed to provide evidence-based information to determine which technique is superior from a patient-centered perspective. We are concerned that these results could be misleading to young cardiac surgeons seeking to undertake minimally invasive aortic valve surgery. This paper is an essential addition to the literature on the topic, but its conclusion may negatively impact uptake of this approach.

Our first concern with this paper is that this study spans nearly 20 years, and Figure E1 shows a decrease in the number of right anterior thoracotomy (ART) cases over time. In other words, most issues of ART collected for propensity score matching are from earlier cases than those of MS, implying that patients in these 2 groups may have undergone surgery at different time frames. We congratulate the authors for trying to capture the learning curve as well, but this may have had more of an influence on the ART outcomes rather than the MS approach.

The ART outcomes are better than the sternotomy approach in a high-volume center.^2^ Meanwhile, our single-surgeon study revealed that the ART and the MS have similar early outcomes but are better than the full sternotomy outcomes.^3^ We also know that the ART approach is likely to be more affected by the operator’s influence, as already seen in mitral surgery.^4^ Some surgeons are better at an MS approach than persevering with an ART approach, which requires specialized instruments and alternative cannulation strategies.

We also wonder whether the difference in survival may be due to different patients or procedures, as the incision alone should not affect survival once the underlying problem is addressed. We assume that patients with similar clinical conditions, such as aortic stenosis, should not have noticeable differences in survival outcomes. Any differences may mask other secondary differences not captured by the propensity matching.

Finally, this paper captures the experience of 5 surgeons, and the message may be that this approach, when introduced into an institution, needs to be closely monitored for outcomes. If there is a deviation from the expected results, the specific surgeon may best move back to another approach, like MS. The ART approach is a good step toward complete endoscopic aortic valve surgery, which has even more potential advantages.^5^ Until a randomized control trial is carried out, trained surgeons who closely monitor their outcomes in real-time are the best to offer this approach.
